# Identification and Quantification of Key Phytochemicals, Phytohormones, and Antioxidant Properties in *Coccinia grandis* during Fruit Ripening

**DOI:** 10.3390/antiox11112218

**Published:** 2022-11-10

**Authors:** In Young Lee, Nami Joo

**Affiliations:** Department of Food and Nutrition, Sookmyung Women’s University, Seoul 04310, Korea

**Keywords:** *Coccinia grandis*, phytochemicals, polyphenols, phytohormones, fruit ripening, antioxidant properties

## Abstract

*Coccinia grandis* contains secondary metabolites, such as flavonoids, phenolic acids, terpenoids, alkaloids, sterols, and glycosides, which are known to have in vitro antioxidant, antidiabetic, anti-inflammatory, and antidyslipidemic activities. *C. grandis* fruits change dramatically during ripening, and the differences in the phytochemicals contribute to various uses. This study reports the phytochemical compounds and antioxidant activities during ripening of *C. grandis* for the first time. Characterizations were conducted on the physiologically active substances in *C. grandis* fruits at three ripening stages, and a total of 25 peaks were identified. Key phytochemicals in the ripening stages of *C. grandis* were identified, and the major substances that contributed to antioxidant properties were selected and quantitatively analyzed. Although the concentration of tiliroside increased during aging, hydroxycinnamic acid (chlorogenic and p-coumaric acids), flavonols (rutin), and triterpenes (cucurbitacins B and D) with antioxidant effects decreased. Therefore, phenolic compounds and cucurbitacins dominate immature *C. grandis* quantitatively. Regarding phytohormones, the gibberellin A4 content decreased as the fruits matured, but indoleacetic acid and salicylic acid increased with fruit maturity. The antioxidant capacities determined by DPPH and ABTS consistently decreased with increasing maturity. Accordingly, the extracts of immature *C. grandis* fruits have high levels of bioactive compounds and can be used to develop food additives and health supplements.

## 1. Introduction

*Coccinia grandis* (*C. grandis*) belongs to the Cucurbitaceae family and has been cultivated in many tropical and subtropical countries. *C. grandis* is a dioecious plant that grows in vines on trees, fences, or supports and has separate male and female flowers. The oval-shaped fruits of *C. grandis* are green when immature and red when fully ripe. *C. grandis* has been considered as a weed and a widespread breed in some parts of Australia and the United States [1]. However, it has been used for culinary and medical purposes in Southeast Asia for a long time and has been cultivated in Korea in recent years owing to climate changes. The phytochemical investigations of *C. grandis* in previous studies have revealed that it contains secondary metabolites, such as flavonoids, phenolic acids, terpenoids, alkaloids, sterols, and glycosides, which are known to have in vitro antioxidant, antidiabetic, anti-inflammatory, and antidyslipidemic activities [2,3,4,5]. Triterpenoid cucurbitacin contained in *C. grandis* is a physiologically active functional ingredient with broad therapeutic significance and is useful for a wide range of biological activities [6]. In addition, the phenolic compounds, including large amounts of phenolic acids and flavonoids, have high antioxidant capacity, providing protection against carcinogenesis, inflammation, atherosclerosis, and thrombosis [7,8,9]. Phytohormones are known to assist growth and development of the plant. However, a recent study suggests that phytohormones also work in humans against various diseases; the phytohormones that affect humans are accrued either through ingestion of the raw plant material or through a processed diet [10]. The amounts and categories of phenolic compounds, cucurbitacins, and phytohormones present in plants are different for each part, such as fruits, leaves, stems, seeds, and skins, but may vary depending on maturity and climatic conditions. The biochemical, physiological, and metabolic changes with fruit ripening lead to variations in the antioxidant properties and bioactive composition, and these circumstantial processes are related to the ultimate quality of the fruit. [11]. Given the biological significance and commercial value of fruits, several phytochemical analyses of the mechanisms underlying fruit ripening have been attempted in recent decades [12,13,14]. The fruits of *C. grandis* change dramatically throughout the process of fruit ripening, and the variations in the phytochemicals contribute to different uses. Further, differences in the nutritional values between the ripening stages of *C. grandis* are practically unknown, although the fruit is consumed at different stages of ripeness. Previous studies have focused on a few compounds in the leaves and fruit of *C. grandis*, but a comprehensive analysis of the phytochemical compositions throughout fruit ripening has not been attempted until now. To the best of our knowledge, this study reports the phytochemical compounds and antioxidant activities during the ripening stages of *C. grandis* fruits for the first time. Hence, the objectives of this study are to identify and quantify the phytochemical compounds as well as the phytohormones of *C. grandis* fruits at three stages of ripening and to evaluate their antioxidant activities. This study is also expected to help understand the biochemistry of fruit ripening.

## 2. Materials and Methods

### 2.1. Chemicals and Reagents

Chlorogenic acid (≥95%), p-coumaric acid (≥98%), rutin (≥95%), quercetin (≥95%), 2,2′-diphenyl-1-picrylhydrazyl (DPPH), 2,2-azinobis-(3-ehtylbenzothiazoline-6-sulphonic acid)-diammonium salt (ABTS), 6-hydroxy-2,5,7,8-tetramethylchromane-2-carboxylic acid (Trolox) and phytohormones (abscisic acid (≥99%), gibberellic acid (≥90%), gibberellin A4 (≥90%), indoleacetic acid (≥98%), jasmonic acid (≥90%), and salicylic acid (≥99%)) were obtained from Sigma-Aldrich (St. Louis, MO, USA). Quercitrin (≥98%), tiliroside (≥99%), pinoresinol (≥95%), cucurbitacin B (≥98%), cucurbitacin D (≥99%), and cucurbitacin I were purchased from Biofron (La Mirada, CA, USA). 

### 2.2. Plant Materials

The fruits of *C. grandis* grown in Yongin-si, Gyeonggi-do (37°11′17.0″ N, 127°18′39.9″ E), Korea, were harvested from 23 August to 2 October 2021. Fruits at three distinguishable stages of ripening were hand-picked randomly from trees according to the number of days to maturity, color, firmness, and size (about 660 pieces in each ripening stages): green ripening stage (GRS; after 12 ± 2 days from flower wilting, bright green, hard texture, and small size), half ripening stage (HRS; after 12 ± 2 days from GRS, green, firm texture, and full size), and full ripening stage (FRS; 12 ± 2 days from HRS, red, soft texture, and full size). The harvested fruits were washed and dried, and then frozen in a deep freezer (New Brunswik Scientific Co., Buckinghamshire, UK) at −70 °C for 48 h. Thereafter, they were dried with a freeze dryer (MCFD 8508, Operon Eng Co., Seoul, Korea) for 72 h, powdered, and stored in a deep freezer for later use.

### 2.3. Identification of Phytochemicals

The ground, freeze-dried *C. grandis* samples were extracted with 80% aqueous ethanol. Briefly, 0.1 g of *C. grandis* powder was extracted with 1 mL of solvent and vortexed for 1 min. Then, the mixture was sonicated for 1 h and filtered with a 0.2 μm membrane filter. *C. grandis* extracts were applied in an Ultimate 3000 ultra-high-performance liquid chromatography (UHPLC) system (Thermo Fisher Scientific Inc., Sunnyvale, CA, USA) to identify the phytochemicals according to ripening stages. Chromatographic separations of the metabolites were performed using a UPLC T3 column (2.1 mm × 150 mm × 1.6 μm, Waters Co., Milford, MA, USA). The flow rate was set to 0.4 mL/min. A 5 μL aliquot of 1000 ppm of *C. grandis* fruits 80% MeOH extract was injected, and the column oven was set to 45 °C. The mobile phases included 0.1% formic acid in HPLC-grade water (Solvent A) and 0.1% formic acid in acetonitrile (Solvent B). Gradient elution consisted of 0–1 min, 5% B; 1–10 min, 5–30% B; 10–20 min, 30–100% B; 20–24 min, 100% B; 24–25 min, 5% B; and a 1.5 min hold time followed by a 3 min re-equilibration to the starting conditions. Mass spectrometry (MS) and MS/MS detections were conducted with a Triple TOFTM 5600+ operating in the positive and negative-ion electrospray modes. The information-dependent acquisition (IDA) mode was used with the mass range at 100–2000 *m*/*z*. The parameters of the optimized MS were as follows: spray voltage of 5500 V in positive mode and 4500 V in negative mode; ion source gas-1 and gas-250 psi; curtain gas pressure of 35 psi; collision gas pressure of 20 psi; source temperature of 500 °C; and collision energy of 35 ± 15 eV in positive mode and −35 ± 15 eV in negative mode. The phytochemical compounds were identified based on their accurate mass and molecular ion fragmentation pattern using Scafford Elements 2.2.1 and other mass spectral libraries, such as MassBank of North America (MoNA) and NIST (accessed: 15 June 2022).

### 2.4. Quantification of Phytochemicals

*C. grandis* extracts (80% methanol aqueous) were prepared and quantified using a UHPLC Vanquish system. Chromatographic separations were performed using a Cortecs C18 column (50 mm × 2.1 mm × 2.7 μm, Waters). The column temperature was set to 45 °C and injection volume was 1 μL with a flow rate of 0.3 mL/min. The mobile phases included 0.1% formic acid in HPLC-grade water (Solvent A) and 0.1% formic acid methanol (Solvent B). Gradient elution consisted of 0–0.5 min, 5% B; 0.5–5 min, 5–95% B; and a 1.5 min hold time followed by a 3 min re-equilibration to the starting conditions. A Thermo TSQ Altis tandem quadrupole mass spectrometer (Thermo Fisher Scientific Inc., Waltham, MA, USA) equipped with a heated electrospray (H-ESI) ion source operating negative-ion selected reaction monitoring (SRM) mode was employed for the analysis. The SRM mode was used with the mass range at 100–2000 *m/z*. The parameters of the optimized MS were as follows: spray voltage of 3500 V at positive mode and −2500 V at negative mode; sheath gas pressure of 50 arb; aux gas pressure of 10 arb; sweep gas pressure of 1 arb; ion transfer tube temperature of 325 °C; vaporizer temperature of 350 °C. The Trace Finder software (Thermo Fisher Scientific Inc., MA, USA.) was used for data acquisition and processing. The validation data including the range, correlation coefficient (r), slope of the calibration curve, standard deviation, intercept, intercept error, IDL, I-LOQ, LOD, and LOQ are summarized in Table S1.

### 2.5. Quantification of Phytohormones

The preparation of extracts was determined according to a modified method described by Salem, M. A. et al. [15]. The freeze-dried *C. grandis* samples (50 mg) were extracted with 0.8 mL of acidified water (0.1% HCl). After vortexing, the samples were placed on an orbital shaker for 30 min at 4 °C and 1 mL of MTBE was added followed by 15 min of sonication. Thereafter, the samples were centrifuged at 10,000× *g* for 10 min at 4 °C. Then, 0.8 mL of the upper supernatant was dried using a speedvac concentrator at 25 °C. The obtained pellets were resuspended in 100 μL of 50% methanol aqueous solution and immediately subjected to the UHPLC Vanquish system. Analytical UHPLC separation was achieved with a Cortecs C18 column (50 mm × 2.1 mm × 1.6 μm, Waters). The column temperature was set to 45 °C and injection volume was 3 μL with a flow rate of 0.3 mL/min. The mobile phases included 0.1% formic acid in HPLC-grade water (Solvent A) and 0.1% formic acid in acetonitrile (Solvent B). Gradient elution consisted of 0–4 min, 95% A; 4–4.3 min, 60% A; 4.3–4.8 min, 90% A; 4.8–5 min, 95% A; 5–8 min, 95% A; and a 1.5 min hold time followed by a 3 min re-equilibration to the starting conditions. A Thermo TSQ Altis tandem quadrupole mass spectrometer equipped with a H-ESI ion source operating negative-ion SRM mode was employed; mass range at 100–2000 *m/z*. The parameters of the optimized MS were as follows: spray voltage of 3500 V at positive mode and −2500 V at negative mode; sheath gas pressure of 50 arb; aux gas pressure of 10 arb; sweep gas pressure of 1 arb; ion transfer tube temperature of 325 °C; vaporizer temperature of 350 °C. The Trace Finder software (Thermo Fisher Scientific Inc., MA, USA.) was used for data acquisition and processing.

### 2.6. Antioxidant Properties

The 2,2-diphenyl-1-picrylhydrazyl (DPPH) radical scavenging ability was measured with reference to the study by Braca et al. [16]. After concentration and freeze drying, the powdered extract sample was mixed with 99.9% ethanol to prepare solutions with different concentrations (1, 5, 25, 50, and 100 ppm). To 2 mL of each concentration solution, about 2 mL of 0.2 mM DPPH ethanolic solution was added, stirred, and left in the dark for 30 min; absorbance was then measured at 515 nm, and the value of the DPPH radical scavenging activity was calculated to the following formula: scavenging rate (%) = [1 − (A1 − A2)/A0] × 100, where A0 was the absorbance of the control, A1 was the absorbance in the presence of the sample and DPPH, and A2 was the absorbance of the sample blank.

The radical scavenging activity of 2,2′-azino-bis-3-ethylbenzothiazoline-6-sulfonic acid (ABTS^•+^) was measured according to the method of Re et al. [17]. After concentration and freeze drying, the powdered extract sample was mixed with 99.9% ethanol to prepare solutions with different concentrations (1, 5, 25, 50, and 100 ppm). A 7 mM ABTS solution and a 2.4 mM potassium persulfate solution, each dissolved in distilled water, were mixed and reacted in a dark place for 12 to 16 h to obtain the ABTS radicals. The absorbance of radical-generated solution was measured at 734 nm and the value was set at 0.70 ± 0.02. After mixing 3.6 mL of the ABTS solution with 0.4 mL of the powdered extract sample, incubation was performed in a dark place for 30 min and absorbance was measured at 734 nm. The results were expressed in mM Trolox per g of *C. grandis* fruit extract.

### 2.7. Statistical Analysis

Raw chromatographic data for identification of key phytochemicals were processed using Scafford Elements version 2.2.1 with automatic peak detection. Mass spectrum de-convolutions were performed with references to the NIST (2017), Human Metabolome Database (HMDB, http://www.hmdb.ca: accessed on 15 June 2022), and MoNA. The principal component analysis (PCA) was performed using SIMCA version 17.0 (Umetrics, Umeå, Sweden). Data were presented as mean and standard deviation (SD). The variance was analyzed using one-way analysis of variance (ANOVA) and the differences between the sample means were analyzed by Duncan’s test at a significance level of 0.05.

## 3. Results and Discussion

### 3.1. Phytochemical Composition

Plant-derived bioactive compounds exert variable biological effects in vitro and in vivo. Among them, the antioxidant properties of plant extracts are probably the most extensively studied, and polyphenols and terpenoids are considered to contribute to these activities representatively. In this study, the UHPLC system was used to gain insights into the qualitative profiles of the chemical compositions of the prepared *C. grandis* fruit extracts. UHPLC–TripleTOF–ESI–MS/MS facilitates the identification of unknown compounds on the basis of their molecular formula, MS/MS fragmentations, and exact mass measurements [18,19]. In addition, UHPLC–TripleTOF–ESI–MS/MS provides separation and targeted fragmentation of particular ions, which may contribute to isomer distinction and structural elucidation [18,19,20]. Analyses of the phytochemicals were reported in the positive and negative ionization modes (Figures S1 and S2). Table 1 shows the results of the phytochemical compounds of *C. grandis* fruit extract according to its ripening stages. In total, 25 different compounds were tentatively identified: 7 hydroxycinnamic acids, 9 flavonols, 1 lignan, 3 triterpenes, and 5 phytohormones.

**Figure 1:**
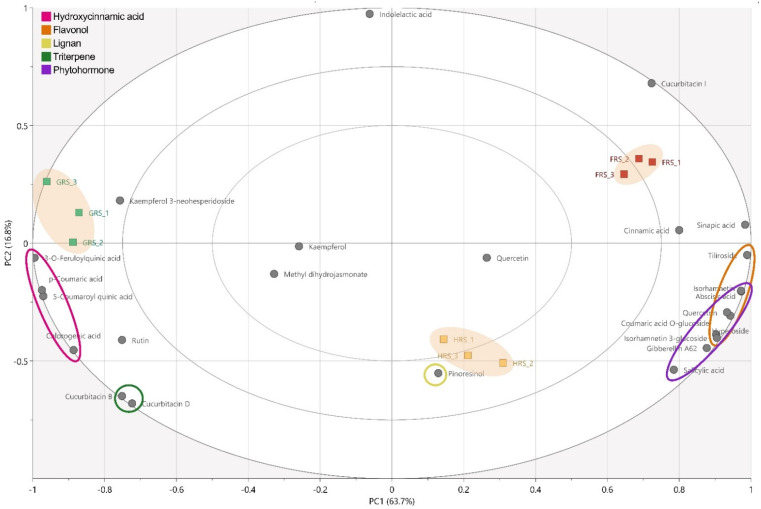
PCA biplot obtained from LC–MS data of *Coccinia grandis* fruits at different stages of ripening.

The multivariate data analyses were conducted on the identified metabolites of *C. grandis* to understand how the differences in the three ripening stages could be indicated and correlated with the phytochemical characteristics. The metabolite fingerprinting of the three different stages of *C. grandis* fruits was carried out by PCA to efficiently visualize the dissimilarities based on chromatographic patterns. The PCA results show distinct separations between the three stages of *C. grandis* fruits. The PCA score plots (Figure S4) representing the analyses of the positive and negative ionization modes describe 80.5% of the total variance in which optimal segregation was achieved between principal component 1 (PC1, 63.7%) and principal component 2 (PC2, 16.8%), where PC1 was the key component for sample separation. The loading plots (Figure S4) and biplot (Figure 1) of the PCA results were consistent with the respective score plots, in which the significant intensities of each of the metabolite variables for their correlative clusters reflected the variations in their metabolite fingerprints. Based on PC1 in the loading plots, the GRS showed higher amounts of hydroxycinnamic acids (chlorogenic acid, 5-coumaroyl quinic acid, p-coumaric acid, 3-*O*-feruloylquinic acid), flavonols (rutin, kaempferol 3-neohesperidoside), and triterpenes (cucurbitacins D and B) than the others (Figure S4). On the other hand, phytohormones (abscisic acid, salicylic acid, and gibberellin A62), tiliroside, and some hydroxycinnamic acids (coumaric acid *O*-glucoside, sinapic acid, and cinnamic acid) were more prevalent in the FRS than the others. The biplot of the PCA results also shows the distribution of the markers in the three stages of *C. grandis* fruits (Figure 1). The preferential distribution of the flavonoids in the first quadrant of the biplot primarily accounts for the differences in the FRS. Moreover, the principal hydroxycinnamic acids, flavonols, and triterpenes distributed in the second quadrant of the biplots account for the variations in the GRS. Furthermore, pinoresinol and quercetin were distributed in the fourth quadrant of the biplots in the HRS. This indicates that variations in the metabolite fingerprints affect the phytochemical distributions according to the ripening stages of *C. grandis* fruits. 

### 3.2. Quantification of the Phytochemicals

In this study, the key phytochemicals according to the ripening stages of *C. grandis* fruits were identified, and the major substances that could potentially affect to the antioxidant properties were selected and quantitatively analyzed. Thus, a total of seven phenolic substances, three triterpenes, and six phytohormones were quantified (Table 2, Figure S3).

#### 3.2.1. Hydroxycinnamic Acids

Phenolic acids, which can be found in many plant species, are phenylpropanoids with an aromatic ring attached to a three-carbon side chain. Many biological activities, such as antioxidant, antimicrobial, antihepatotoxic, antiosteoporotic, antiulcer, immunomodulatory, and apoptotic activities, are attributed to phenolic acids [21,22,23,24,25,26,27]. One class of phenolic acids—hydroxycinnamic acids—is greatly abundant in food and regarded to account for about one-third of dietary phenolic compounds [8]. Several hydroxycinnamic acids and their glycosides, such as chlorogenic acid, coumaric acid, and ferulic acid, are mainly found in medicinal food plants of the Cucurbitaceae family [6]. During the ripening process, the individual hydroxycinnamic acids were found to be available in different amounts. The highest concentrations of the individual hydroxycinnamic acids were generally found in immature *C. grandis* fruits and decreased during subsequent ripening. Chlorogenic acid was quantitatively the most dominant hydroxycinnamic acid while p-coumaric acid was found in lower amounts. Chlorogenic acid is one of the most abundant polyphenols in the human diet and is formed by esterification of quinic and caffeic acids [28]. Results of in vivo and in vitro experiments show that chlorogenic acid mostly has important antioxidant and anticarcinogenic activities [29]. In general, our results showed that the quantities of the two phenolic acids decreased during ripening, particularly chlorogenic acid, which is known to for its antioxidant properties. This indicates the important role of hydroxycinnamic acids in antioxidant metabolism during the ripening of *C. grandis* fruits.

#### 3.2.2. Flavonols

Flavonoids, including flavonols, have high antioxidant capacities and play protective roles in inflammation, atherosclerosis, carcinogenesis, diabetes, and thrombosis [7,8,9]. Moreover, flavonoids interact with various enzymatic systems; their inhibition of the enzymes lipooxygenase and cyclooxygenase lead to protection against cardiovascular diseases, anti-inflammatory activities, and cancer chemoprevention [13,14,16,17,18]. The amount of rutin as a flavonol was most abundant in *C. grandis* at the immature stage, and this content decreased remarkably during ripening. Meanwhile, the amount of quercetin increased gradually with ripening. Quercetin has the capacity to ameliorate the autoimmune disorder encephalomyelitis, which is related to immune responses mediated via Th1 cells [30]. Quercitrin is a glycoside derivative of the flavonoid quercetin and the de-oxy sugar rhamnose. Trace amounts of quercitrin were confirmed in *C. grandis* fruits, and the highest content was observed at HRS. Tiliroside, also known as kaempferol 3-*O*-glucoside-6”-E-coumaroyl, is widely used as food and medicine and has been applied in the treatment for various ailments [31]. Tiliroside exerts antioxidant, antiobesity, antidiabetic, anticarcinogenic, and hepatoprotective effects [32]. In the present study, tiliroside showed a remarkably high content at the ripening stage. In *C. grandis*, rutin decreased drastically throughout the fruit ripening process, while tiliroside increased dramatically, which resulted in differences in color, texture, and flavonoid content between the immature and mature fruits. These differences determine their uses. Previous epidemiological studies have suggested an inverse relationship between the intake of foods high in flavonoids and incidence of various diseases [33]. Notably, quercetin and rutin may be closely related to benefits to the consumers [34]. Therefore, immature fruits are particularly rich in flavonoids, which could be functional in the development of healthcare foods, while the mature fruits with a less astringent taste can be used as agricultural products.

#### 3.2.3. Lignan

Recently, lignans have emphasized for their importance among the various dietary phytochemicals because of their possible effects on human health [35]. Lignans belong to phenylpropanoid family of organic compounds and express enormous structural diversity and biological activities [36]. Several studies have reported that intakes of abundant lignan have biological benefits, such as antioxidant, antitumor, antiviral, antibacterial, and antiplatelet activities, in addition to protective effects against coronary heart diseases [37,38]. Among the plant lignans, only pinoresinol was identified in *C. grandis* fruits. Pinoresinol has strong anti-inflammatory properties by blocking the NF-kB signaling pathway in relation to conjugation efficacy in intestinal cells [37]. Interestingly, our results showed that pinoresinol increased until the HRS, but decreased thereafter in the FRS.

#### 3.2.4. Triterpenes

Plants belonging to the Cucurbitaceae family have medicinal significance due to the presence of phytochemicals such as tannins, glycosides, saponins, carotenoids, phytosterols, and most importantly, the triterpene cucurbitacin. The most common cucurbitacins in the Cucurbitaceae family are types B and D [39]. In previous studies, cucurbitacin was reported to have anti-inflammatory, antiangiogenic, immunomodulatory, cytotoxic, cytostatic, and hepatoprotective properties in in vitro and in vivo models [40,41]. In particular, cucurbitacin B was shown to have strong antitumor activity and effectiveness against inflammation and chronic hepatitis [39] and showed efficacy in inhibiting lung cancer, breast cancer, and colon cancer cell lines. In addition, cucurbitacin D is valuable as an antitumor drug that induces apoptosis in human-derived liver tumor cells [42] and has immunomodulatory properties that induce inflammasomes in macrophages [43]. From the quantitative analyses of cucurbitacins B and D in *C. grandis* fruits, it was found that both decreased as the fruit matured. The amount of cucurbitacin B was found to be large in the GRS, but only a trace amount remained in the FRS. In addition, the content of cucurbitacin D was highest in the GRS, suggesting that the immature *C. grandis* fruit has pharmacological potential and has potential as a source of functional foods and therapeutic agents. Previous studies have reported that cucurbitacin E was found in *C. grandis* fruits [44]. However, in this study, cucurbitacin B was found at all stages, cucurbitacin D was identified in the GRS and HRS, and cucurbitacin I was found in trace amounts in the HRS. 

#### 3.2.5. Phytohormones

Phytohormones have long been known to be innately associated with fruit development and ripening [45,46]. Recently, phytohormones have been considered as an alternative to chemicals in enhancing the nutritional value and supporting the high quality of agricultural products [47]. Further, phytohormones have an influence on biological activities such as metabolic activity and disease resistance in plants. An inadequately known fact is that animals produce and are affected by phytohormones [48]. Phytohormones have considerable roles in humans and are accumulated through processed diets and intake of raw plant material. Therefore, a high concentration of phytohormones in the human body is linked to a number of physiological and metabolic responses. These phytohormones affect antioxidant response, glucose metabolism, cellular processes, cell division, cell cycle regulation, inflammation, cancer, etc. [49,50,51].

Four kinds of phytohormones found in the identification (abscisic acid (ABA), salicylic acid (SA), indolelacetic acid (IAA), gibberellin A) were quantitatively analyzed. In addition, jasmonic acid (JA), a precursor of methyl dihydrojasmonate, was also quantified (Table 3). It was found that the content of ABA decreased first and then increased as the *C. grandis* fruit matured. Human beings are constantly exposed to ABA; the daily consumption of fruits and vegetables in our diet leads to accumulation of ABA in the body [52]. The adipose tissues of humans release ABA at high and low glucose levels. In hyperglycemia, the release of GLP-1 induces the β cells and insulinoma cells to release ABA and insulin, which promote glucose uptake in skeletal muscles and adipocytes. Accordingly, several studies have reported that glucose homoeostasis is regulated by ABA [53]. Moreover, ABA also operates as a proinflammatory endogenous cytokine as it stimulates phagocytosis, nitric oxide (NO) and reactive oxygen species (ROS) production, chemotaxis, and chemokinesis, thereby inducing the innate immunity [48]. Thus, the intake of ABA-rich foods could improve our innate immune system and prevent metabolic disorders such as type 2 diabetes. On the other hand, the content of GA4 decreased but IAA and SA tended to increase as the fruits matured. The SA content in particular increased significantly. Gibberellic acids (GAs) are involved mainly in fruits and stem growth [54]. GA in the human body primarily comes from a plant-based diet. It was noted that GAs inhibited the formation of free radicals [55] and hepatotoxicity in albino rats as potent pro-oxidants [56]. JA and SA regulate secondary metabolites and multiple phytochemicals, thereby serving as signal molecules in several physiological processes [57].

## 4. Antioxidant Activities

The antioxidant activity was shown as a decrease in absorbance according to DPPH radical scavenging. The concentration of the antioxidants required to reduce absorbance by 50% for 0.2 mM DPPH radical activity was expressed as the IC50 value, and as IC50 increased, the antioxidant activity decreased. The IC50 value decreased in proportion to the concentration of the *C. grandis* extract, and it was found that the *C. grandis* extract has a positive effect on the DPPH free radical scavenging activity. The values of IC50 were 5.87 μg/mL in GRS, 29.88 μg/mL in HRS, and 83.97 μg/mL in FRS (Table 4). As expected, the antioxidant activities decreased as the fruits matured. The sum of the individual phytochemical compounds in immature fruits is four times higher than that at full ripeness. It was confirmed, nevertheless, that all *C. grandis* fruit extracts generally showed promising antioxidant activities, which is effective for scavenging various free radicals generated by oxidative stress in the living body. ABTS radical scavenging activity can be considered as a mechanism for reducing oxidation of ABTS with potassium persulfate by the presence of hydrogen-donating antioxidants. [17]. The ABTS radical scavenging activities of *C. grandis* were 2.09 mM Trolox/g in GRS, 1.25 mM Trolox/g in HRS, and 0.70 mM Trolox/g in FRS, suggesting that GRS had the best ABTS free radical scavenging ability. It was observed that the reduction in the antioxidant capacity over the course of maturity, especially between unripe and intermediate ripeness, was less distinct for ABTS than for DPPH. The antioxidant potential of *C. grandis* fruits could be attributed to their high phytochemical profiles as the antioxidant properties of the main groups of compounds (hydroxycinnamic acids, flavonols, lignans, triterpenes, and phytohormones) have been studied in depth. Our results suggest that the ripening processes in *C. grandis* fruits may affect the content of the phytochemical compounds and their antioxidant activities. However, further studies related to enzymes for biosynthesis of phytochemicals during ripening of *C. grandis* and expression of the genes encoding these enzymes would be necessary for understanding and explaining the metabolic changes in these compounds.

## 5. Conclusions

The chemical characterizations of *C. grandis* fruits at three stages of maturity showed significant differences in all determined parameters. In this study, the identifications were conducted to confirm the physiologically active substances in *C. grandis* fruits based on maturity, and a total of 25 peaks were identified. These 25 peaks showed significant differences in terms of the chemical properties of *C. grandis* fruits at three ripening stages. The key phytochemicals according to the ripening stage of the fruits were identified, and the major substances among them that contribute to antioxidant properties were selected and quantitatively analyzed. Based on the results, although the concentration of tiliroside increased during the aging process, hydroxycinnamic acids (chlorogenic acid and p-coumaric acid), flavonols (rutin), and triterpenes (cucurbitacins B and D) with antioxidant effects tended to decrease. Therefore, it was determined that phenolic compounds and cucurbitacins found in immature *C. grandis* quantitatively dominate the composition. For the phytohormones, it was found that the content of GA4 decreased but IAA and SA tended to increase significantly as the fruit matured. The antioxidant capacity values determined by DPPH and ABTS consistently decreased with increasing maturity. Accordingly, it is thought that the extracts of immature fruits of *C. grandis* have a high content of bioactive compounds and can be used as innovative materials to develop food additives and health supplements. The information in this study can be useful for providing the functional roles of the identified phytochemical compounds that can contribute to the prevention of various health disorders. Further studies would be necessary to evaluate the bioabsorption, biodisposal, and interactions between the compounds in *C. grandis* fruits after consumption. 

## Figures and Tables

**Table 1:** Identification of the phytochemicals and phytohormones of *Coccinia grandis* fruits at different stages of ripening.

#	Analyte Name	RT (min)	Molecular Formula	Molecular Weight	Adduct	*m/z*	Error (ppm)		
							GRS	HRS	FRS
	Hydroxycinnamic acids								
1	Coumaric acid *O*-glucoside	4.53	C_15_H_18_O_8_	326.1002	[M + HCOO]^−^	371.0984	0.295	0.173	0.315
2	Chlorogenic acid	7.09	C_16_H_18_O_9_	354.0951	[M − H]^−^	353.0878	0.159	0.090	0.053
3	Sinapic acid	8.69	C_11_H_12_O_5_	224.0685	[M + H]^+^	225.0757	0.859	0.737	0.899
4	5-Coumaroyl quinic acid	10.66	C_16_H_18_O_8_	338.1002	[M − H]^−^	337.0929	0.113	0.012	0.095
5	p-Coumaric acid	10.72	C_9_H_8_O_3_	164.0473	[M + H − H_2_O]^+^	147.0441	0.758	1.745	1.561
6	3-O-Feruloylquinic acid	12.99	C_17_H_20_O_9_	368.1107	[M + H]^+^	369.1180	0.553	0.294	0.251
7	Cinnamic acid	30.87	C_9_H_8_O_2_	148.0524	[M + H]^+^	149.0597	1.199	1.270	0.139
	**Flavonols**								
8	Rutin	15.94	C_27_H_30_O_16_	610.1534	[M − H]^−^	609.1461	0.628	0.139	0.017
9	Quercetin	16.14	C_15_H_10_O_7_	302.0427	[M + H]^+^	303.0499	1.671	1.708	1.623
10	Hyperoside	16.20	C_21_H_20_O_12_	464.0955	[M − H]^−^	463.0882	0.184	0.224	0.343
11	Kaempferol 3-neohesperidoside	17.05	C_27_H_30_O_15_	594.1585	[M − H]^−^	593.1512	0.382	0.200	0.107
12	Kaempferol	17.28	C_15_H_10_O_6_	286.0477	[M + H]^+^	287.0550	1.754	2.019	1.913
13	Quercitrin	17.33	C_21_H_20_O_11_	448.1006	[M − H]^−^	447.0933	0.091	0.002	0.890
14	Isorhamnetin 3-glucoside	17.68	C_22_H_22_O_12_	478.1111	[M − H]^−^	477.1038	0.177	0.22	0.716
15	Tiliroside	20.80	C_30_H_26_O_13_	594.1373	[M − H]^−^	593.1301	0.145	0.152	0.428
16	Isorhamnetin	22.86	C_16_H_12_O_7_	316.0583	[M − H]^−^	315.0510	-	0.304	0.197
	**Lignan**								
17	Pinoresinol	19.20	C_20_H_22_O_6_	358.1416	[M + H − H_2_O]^+^	341.1384	1.196	1.643	1.261
	**Triterpenes**								
18	Cucurbitacin I	15.40	C_30_H_42_O_7_	514.2930	[M + H − H_2_O]^+^	497.2898	-	-	1.105
19	Cucurbitacin D	24.09	C_30_H_44_O_7_	516.3087	[M + H − H_2_O]^+^	499.3054	0.891	1.282	-
20	Cucurbitacin B	27.83	C_32_H_46_O_8_	558.3193	[M + HCOO]^−^	603.3175	0.120	0.282	-
	**Phytohormones**								
21	Salicylic acid	1.80	C_7_H_6_O_3_	138.0317	[M − H]^−^	137.0244	0.637	0.653	0.857
22	Indoleacetic acid	4.12	C_10_H_9_NO_2_	175.1839	[M + H − H_2_O]^+^	188.0706	0.134	0.210	0.483
23	Abscisic acid	6.87	C_15_H_20_O_4_	264.1362	[M + H]^+^	264.1434	0.723	0.978	0.925
24	Gibberellin A62	16.01	C_19_H_22_O_5_	330.1467	[M + HCOO]^−^	375.1449	0.124	0.330	0.943
25	Methyl dihydrojasmonate	22.05	C_13_H_22_O_3_	226.1569	[M + H]^+^	227.1642	1.489	1.124	1.220

Each compound was identified by comparison with the database; NIST, HMDB, and MoNA export LC–MS, MS/MS Library.

**Table 2:** Changes in the phytochemical compositions (mg/kg) of *Coccinia grandis* fruits to the dry matter at different stages of ripening.

Compounds	GRS	HRS	FRS	F-Value
**Hydroxycinnamic acids**				
Chlorogenic acid	56.13 ± 2.29 ^a^	25.18 ± 0.09 ^b^	9.96 ± 0.18 ^c^	940.51 ***
p-Coumaric acid	8.20 ± 0.81 ^a^	2.74 ± 0.21 ^b^	2.46 ± 0.03 ^b^	134.13 ***
**Flavonols**				
Quercetin	0.64 ± 0.01 ^c^	1.84 ± 0.01 ^b^	2.03 ± 0.03 ^a^	4529.71 ***
Quercitrin	0.52 ± 0.02 ^b^	1.57 ± 0.02 ^a^	0.44 ± 0.01 ^c^	4915.79 ***
Rutin	152.40 ± 0.57 ^a^	146.45 ± 4.91 ^b^	114.12 ± 0.63 ^c^	153.89 ***
Tiliroside	3.49 ± 0.03 ^c^	50.86 ± 0.88 ^b^	85.97 ± 0.13 ^a^	19,439.95 ***
**Lignan**				
Pinoresinol	2.99 ± 0.00 ^c^	6.00 ± 0.02 ^a^	3.17 ± 0.02 ^b^	34,645.58 ***
**Triterpenes**				
Cucurbitacin B	660.81 ± 11.88 ^a^	340.21 ± 7.35 ^b^	0.33 ± 0.02 ^c^	5029.44 ***
Cucurbitacin D	25.95 ± 0.76	18.35 ± 0.43	n.d.	227.28 ***
Cucurbitacin I	n.d.	0.01 ± 0.01	n.d.	-

n.d.—not detected; values are given as means ± standard deviation with *n* = 3; different letters indicate the significance of differences (*p* < 0.05); *** indicates significant effects at the level of *p* < 0.001, using one-way ANOVA analysis.

**Table 3:** Changes in the phytohormone compositions (ng/g) in *Coccinia Grandis* fruits to the dry matter at different stages of ripening.

Compounds	GRS	HRS	FRS	F-Value
ABA	205.70 ^b^	113.72 ^c^	265.23 ^a^	172.50 ***
GA3	1.13 ^a^	1.90 ^a^	0.95 ^a^	3.47
GA4	48.89 ^a^	20.58 ^b^	n.d.	11.89 *
IAA	2.29 ^c^	12.20 ^b^	51.90 ^a^	219.92 ***
JA	6.14 ^a^	n.d.	3.21 ^b^	29.18 **
SA	209.69 ^c^	1451.69 ^b^	2879.24 ^a^	3124.91 ***

ABA—abscisic acid; GA3—gibberellic acid; GA4—gibberellin A4; IAA—indoleacetic acid; JA—jasmonic acid; SA—salicylic acid; n.d.—not detected; values are given as means ± standard deviation with *n* = 3; the different letters indicate the significance of differences (*p* < 0.05); *, **, *** indicates significant effects at the level of *p* < 0.05, *p* < 0.01, and *p* < 0.001, respectively, using one-way ANOVA analysis

**Table 4:** Antioxidant properties of *Coccinia grandis* at different stages of ripening.

	GRS	HRS	FRS	F-Value
DPPH ^a^	5.87 ± 0.03 ^c^	29.88 ± 0.19 ^b^	83.97 ± 0.71 ^a^	26,295.98 ***
ABTS ^b^	2.09 ± 0.94 ^c^	1.25 ± 0.50 ^b^	0.70 ± 0.58 ^a^	860.87 ***

a: effective concentration at which 50% of the DPPH radicals are scavenged (IC50, extract concentration (μg/mL)); b: expressed in mM Trolox/g of the *C. grandis* fruit extract. The different letters indicate the significance of the differences (*p* < 0.05); *** indicates significant effects at the level of *p* < 0.001, using one-way ANOVA analysis

## Data Availability

The data are contained within the article and supplementary materials.

## Supplementary Materials

The following supporting information can be downloaded at https://www.mdpi.com/article/10.3390/antiox11112218/s1, Figure S1: Total Ion Chromatogram of methanolic extract of *C. grandis* in positive mode (A) GRS, (B) HRS, (C) FRS; Figure S2: Total ion chromatogram (TIC) of methanolic extract of *C. grandis* in negative mode (A) GRS, (B) HRS, (C) FRS; Figure S3: Chromatogram of standard solution mixtures (A) Polyphenols, (B) Triterpenes, (C) Phytohormones; Figure S4: Score biplot and loading obtained from LC–MS data of Coccinia grandis fruits at different stages of ripening (A) Score plot, (B) Loading plot (PC 1), (C) Loading plot (PC 2); Table S1: Validation data including the range, correlation coefficient (r), slope of the calibration curve, standard deviation, intercept, intercept error, IDL, I-LOQ, LOD, and LOQ for the analytes.
