# Supplementary material for: Identification and Quantification of Key Phytochemicals, Phytohormones, and Antioxidant Properties in Coccinia grandis during Fruit Ripening
Source: Antioxidants (Basel). 2022 Nov 10;11(11):2218. doi: 10.3390/antiox11112218 (PMC9686947; doi:10.3390/antiox11112218)
Supplement: Supplementary file 1 [file antioxidants-11-02218-s001.zip › antioxidants-2007672-supplementary.pdf]

Supplementary Materials:

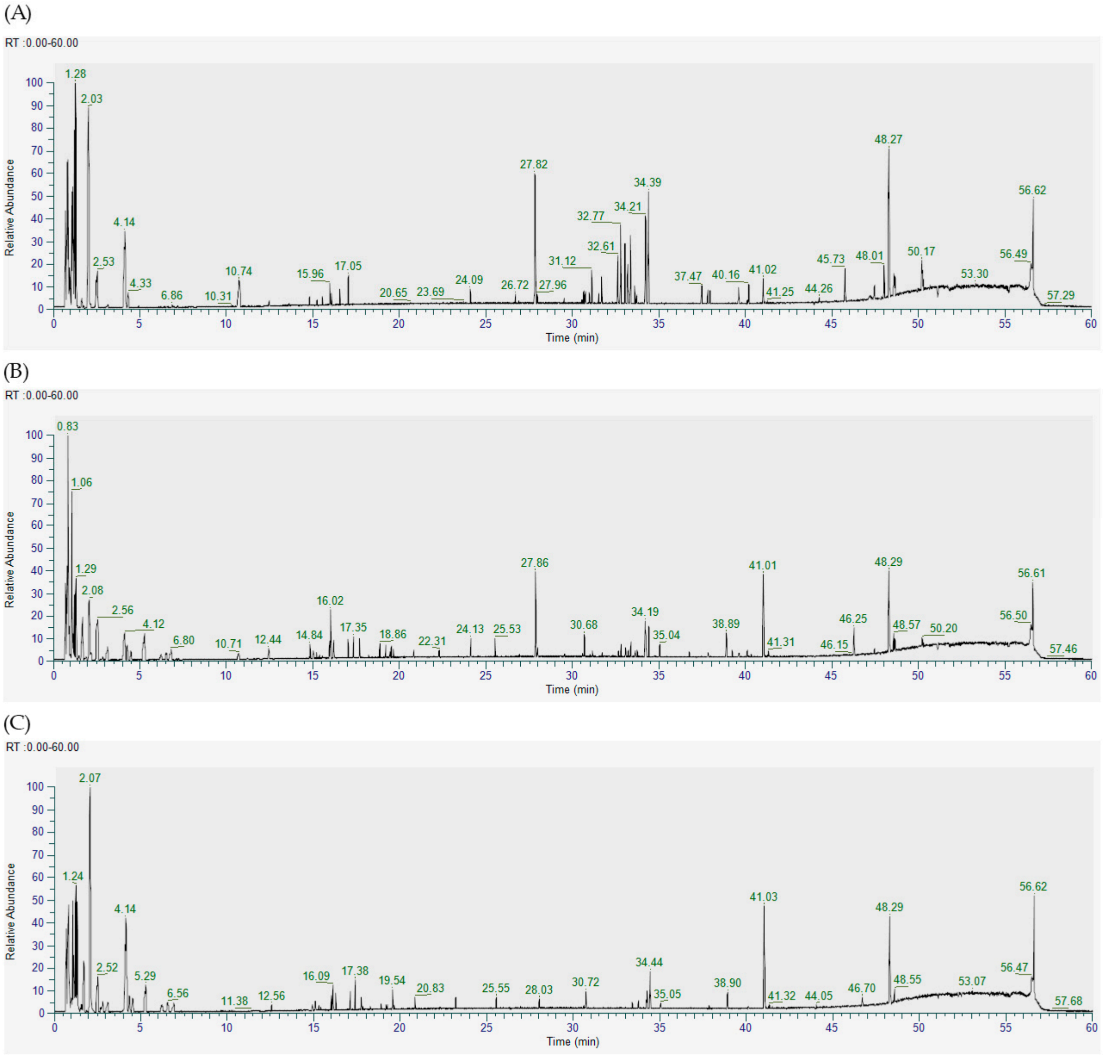

Figure S1. Total Ion Chromatogram of methanolic extract of *C. grandis* in positive mode (A) GRS, (B) HRS, (C) FRS.

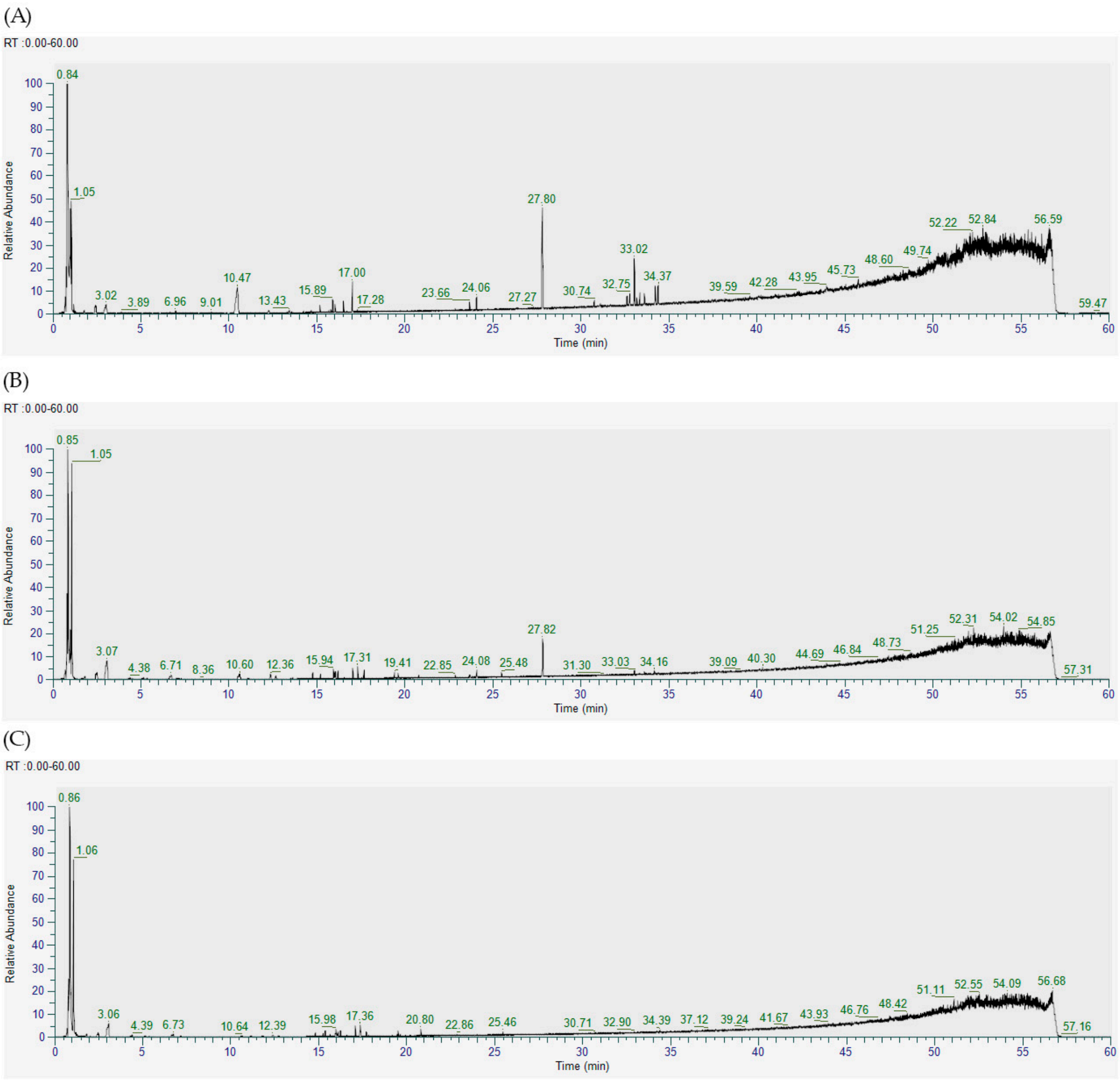

Figure S2. Total ion chromatogram (TIC) of methanolic extract of *C. grandis* in negative mode (A) GRS, (B) HRS, (C) FRS.

(A)

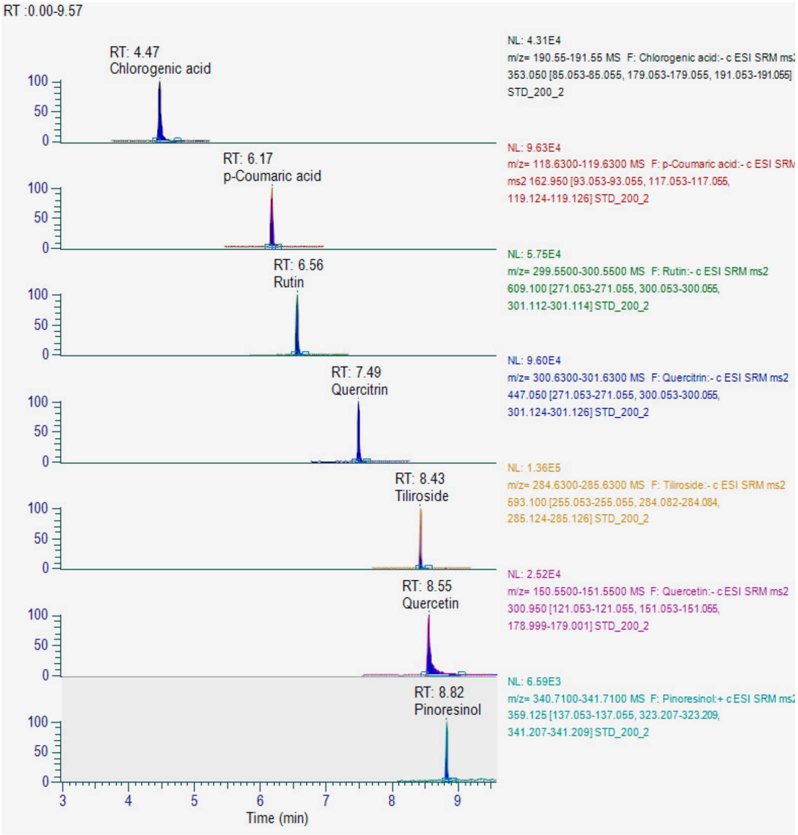
RT :0.00-9.57
RT: 4.47
Chlorogenic acid
NL: 4.31E4
m/z= 190.55-191.55 MS F: Chlorogenic acid:- c ESI SRM ms
353.050 [85.053-85.055, 179.053-179.055, 191.053-191.055]
STD_200_2
RT: 6.17
p-Coumaric acid
NL: 9.63E4
m/z= 118.6300-119.6300 MS F: p-Coumaric acid:- c ESI SRN
ms2 162.950 [93.053-93.055, 117.053-117.055,
119.124-119.126] STD_200_2
RT: 6.56
Rutin
NL: 5.75E4
m/z= 299.5500-300.5500 MS F: Rutin:- c ESI SRM ms2
609.100 [271.053-271.055, 300.053-300.055,
301.112-301.114] STD_200_2
RT: 7.49
Quercitrin
NL: 9.60E4
m/z= 300.6300-301.6300 MS F: Quercitrin:- c ESI SRM ms2
447.050 [271.053-271.055, 300.053-300.055,
301.124-301.126] STD_200_2
RT: 8.43
Tiliroside
NL: 1.36E5
m/z= 284.6300-285.6300 MS F: Tiliroside:- c ESI SRM ms2
593.100 [255.053-255.055, 284.082-284.084,
285.124-285.126] STD_200_2
RT: 8.55
Quercetin
NL: 2.52E4
m/z= 150.5500-151.5500 MS F: Quercetin:- c ESI SRM ms2
300.950 [121.053-121.055, 151.053-151.055,
178.999-179.001] STD_200_2
RT: 8.82
Pinoresinol
NL: 6.59E3
m/z= 340.7100-341.7100 MS F: Pinoresinol:+ c ESI SRM ms
359.125 [137.053-137.055, 323.207-323.209,
341.207-341.209] STD_200_2
Time (min)

(B)

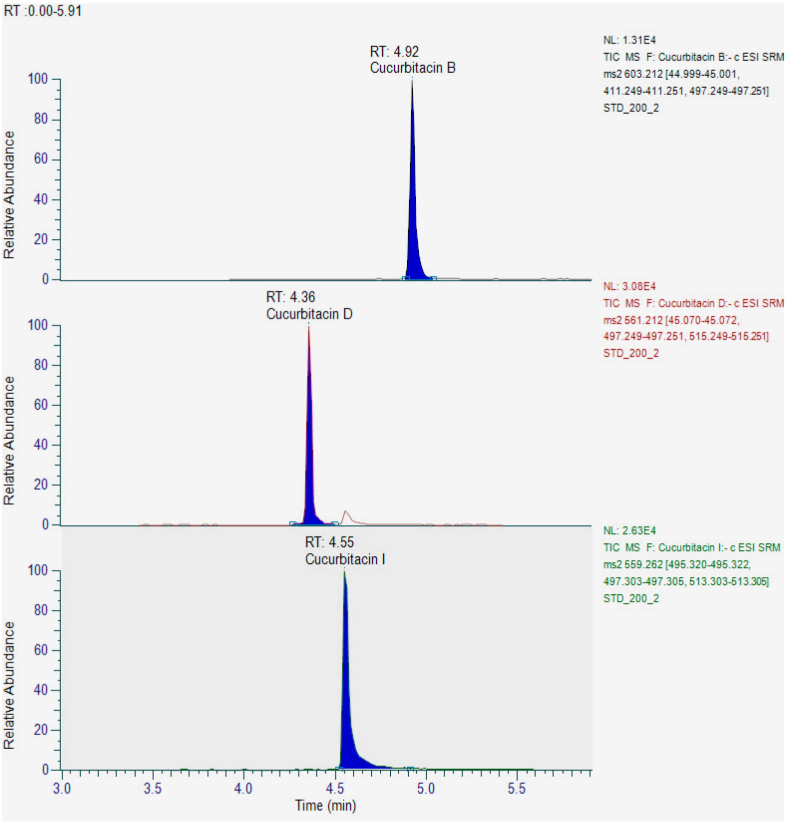
RT :0.00-5.91
RT: 4.92
Cucurbitacin B
NL: 1.31E4
TIC MS F: Cucurbitacin B:- c ESI SRM
ms2 603.212 [44.999-45.001,
411.249-411.251, 497.249-497.251]
STD_200_2
RT: 4.36
Cucurbitacin D
NL: 3.08E4
TIC MS F: Cucurbitacin D:- c ESI SRM
ms2 561.212 [45.070-45.072,
497.249-497.251, 515.249-515.251]
STD_200_2
RT: 4.55
Cucurbitacin I
NL: 2.63E4
TIC MS F: Cucurbitacin I:- c ESI SRM
ms2 559.262 [495.320-495.322,
497.303-497.305, 513.303-513.305]
STD_200_2
Relative Abundance
Time (min)

(C)

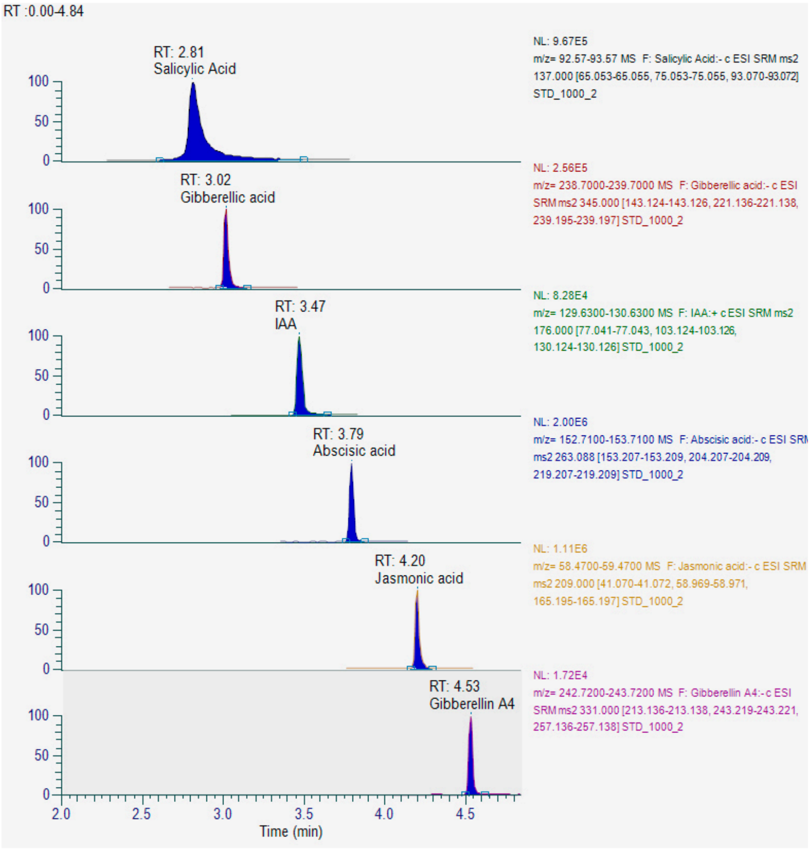

Figure S3. Chromatogram of standard solution mixtures (A) Polyphenols (B) Triterpenes (C) Phytohormones.

(A)

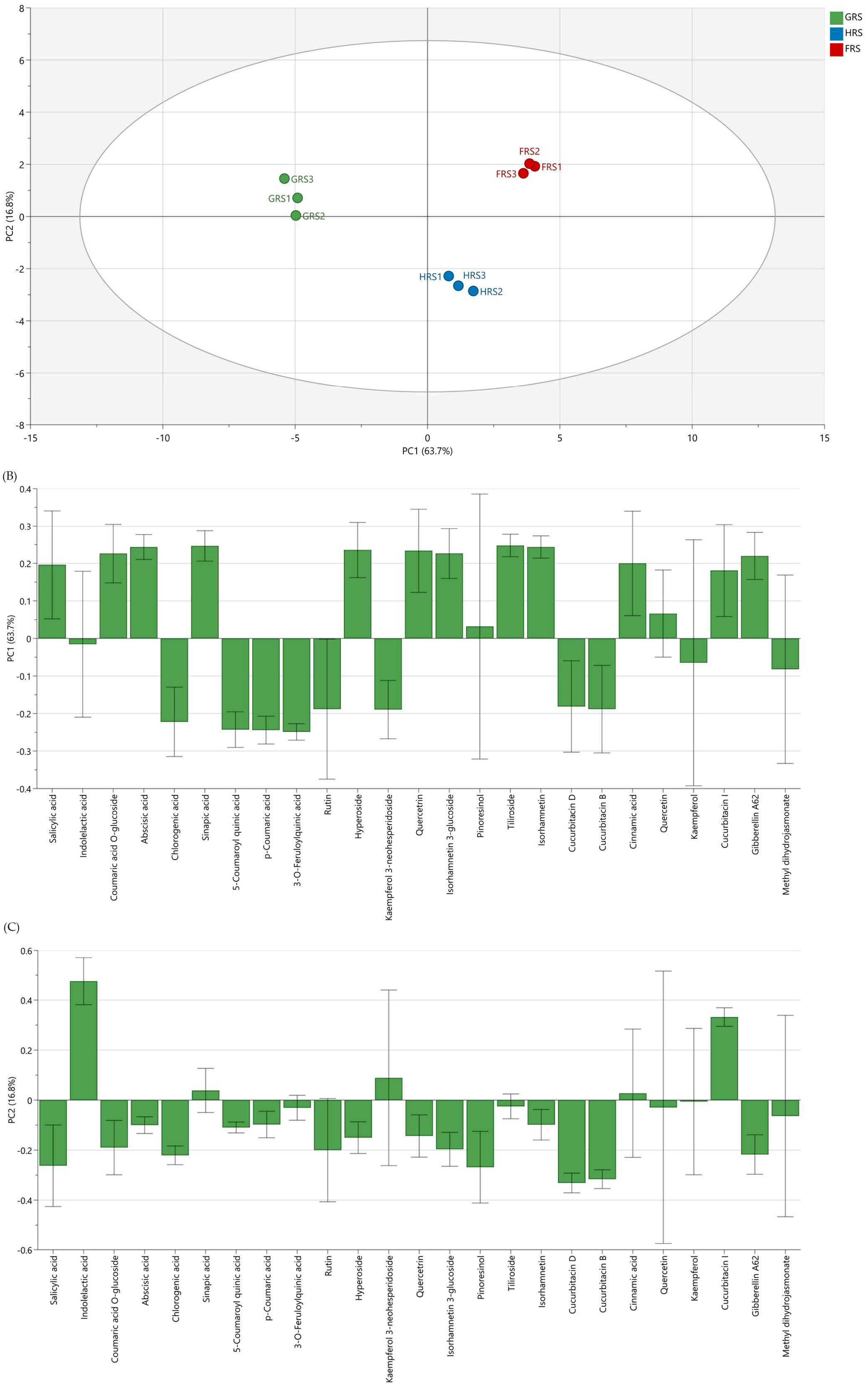

Figure S4. Score biplot and loading obtained from LC-MS data of *Coccinia grandis* fruits at different stages of ripening (A) Score plot, (B) Loading plot (PC 1), (C) Loading plot (PC 2).

Table S1. Validation data including the range, correlation coefficient (r), slope of the calibration curve, standard deviation, intercept, intercept error, IDL, I-LOQ, LOD, LOQ for the analytes**.**

Analyte Name	Range	r2	Slope of the calibration curve (S)	Standard deviation (σ)	Intercept	Intercept error	IDL (ng/mL)	I-LOQ (ng/mL)	LOD = 3.3σ/S	LOQ =10σ/S
**Hydroxycinnamic acids**										
Chlorogenic acid	1−200	0.9999	654.56119	2.98072	−1650.1475	1286.00901	0.01503	0.04554	0.15027	0.45538
p-Coumaric acid	1−200	0.9999	1106.68621	4.23729	−1188.93817	1710.09056	0.01264	0.03829	0.12635	0.38288
**Flavonols**										
Quercetin	1−200	0.9999	459.03227	1.6648	−1242.82828	324.01067	0.01197	0.03627	0.11968	0.36268
Quercitrin	1−200	0.9999	944.86202	2.70439	770.48535	235.384	0.00945	0.02862	0.09445	0.28622
Rutin	1−200	0.9999	583.72238	1.69785	−117.96905	330.44387	0.00960	0.02909	0.09599	0.29087
Tiliroside	1−200	0.9999	1264.20427	3.44481	−1020.57657	1091.05152	0.00899	0.02725	0.08992	0.27249
**Lignan**										
Pinoresinol	5−200	0.9999	57.04133	0.10122	251.61347	43.67068	0.00586	0.01775	0.05856	0.17745
Triterpenes										
Cucurbitacin B	1−1000	0.9999	105.98924	0.18708	196.03561	75.50393	0.00582	0.01765	0.05825	0.17651
Cucurbitacin D	1−1000	0.9999	189.58872	0.25215	329.78722	95.94343	0.00439	0.01330	0.04389	0.13300
Cucurbitacin I	1−1000	0.9999	30.5554	0.0652	−10.55902	23.53632	0.00704	0.02134	0.07042	0.21338
**Phytohormone**										
Abscisic acid	1−100	0.9999	3630.01442	16.39196	−4047.3246	1334.5862	0.01490	0.04516	0.29803	0.45157
Gibberellic acid	1−100	0.9999	739.58361	1.39023	−129.95983	59.98068	0.00620	0.01880	0.12406	0.18797
Gibberellin A4	1−100	0.9999	37.01639	0.41086	1.48927	19.1456	0.03663	0.11099	0.73256	1.10994
Indoleacetic acid	1−100	0.9999	318.69701	1.45715	50.44385	32.74528	0.01509	0.04572	0.30177	0.45722
Jasmonic acid	1−100	0.9999	2031.44078	4.66842	−940.63147	104.90961	0.00758	0.02298	0.15167	0.22981
Salicylic Acid	1−100	0.9999	5014.04266	20.58744	−20757.9842	7431.67977	0.01355	0.04106	0.27099	0.41060
